# Determining optimal transit dosimetry gamma parameter values for the detection of failure modes using receiver operating curve analysis

**DOI:** 10.1002/acm2.70424

**Published:** 2025-12-29

**Authors:** David Sánchez‐Artuñedo, Paula Navarro‐Palomas, Marcelino Hermida‐López, Maria Amor Duch‐Guillén, Mercè Beltran‐Vilagrasa

**Affiliations:** ^1^ Servei de Física i Protecció Radiològica, Hospital Universitari Vall d'Hebron Vall d'Hebron Barcelona Hospital Campus Barcelona Spain; ^2^ Servei d'Oncologia Radioteràpica Hospital Clínic de Barcelona Barcelona Spain; ^3^ Universitat Politècnica de Catalunya Institut de Tècniques Energètiques Barcelona Spain

**Keywords:** PerFRACTION, sensitivity, specificity, transit dosimetry

## Abstract

**Purpose:**

To determine the gamma criteria that maximize the sensitivity and specificity of the transit dosimetry software PerFRACTION (Sun Nuclear Corporation) under five possible failure modes in external beam radiotherapy.

**Methods:**

We simulated five failure modes that potentially introduce large changes in the absorbed dose distribution: (1) Linac hardware incidents. In a VMAT head and neck treatment plan, erroneous plans were created, introducing known errors in the MLC aperture, the collimator angle, and the monitor units. (2) Breathing management protocol incidents. Based on a 4D‐CT of a lung treatment, we created expiration and inspiration CT images. A treatment plan was created using each CT and recalculated in the alternate CT. (3) Patient identification incidents. The treatment plan of one breast patient was recalculated on another patient, and vice versa. (4) Selection of the planning CT incidents. A lung patient had two planning CTs with the presence/absence of lung fluid. A treatment plan was generated for each CT and recalculated for the other. (5) Bolus positioning incidents. An erroneous treatment plan for a breast plan was created without the bolus. For the five failure modes, the transit images were compared using seven gamma criteria, both global and local. Receiver‐operating characteristic (ROC) curves were generated based on the change in the PTV mean dose: > 5%, > 10%, and > 20%. The area under the curve (AUC) and optimal passing rate were calculated.

**Results:**

The global gamma criterion γ(10%/1 mm) maximizes PerFRACTION sensitivity and specificity to detect failure modes that introduce a change in the PTV mean dose exceeding 10%. The standard global gamma criterion, as γ(3%/3 mm), maximizes PerFRACTION sensitivity and specificity to detect deviations in the PTV mean dose above 5%.

**Conclusions:**

A 10%, 1 mm global gamma parameter value produces the needed sensitivity and specificity to identify erroneous deliveries at a level of dose differences of 10% or greater.

## 1 INTRODUCTION

A significant concern in radiotherapy is that the dose calculated by the treatment planning system (TPS) must agree with the dose administered to the patient throughout the treatment process. Ideally, the difference between the administered dose and the dose calculated by the TPS should be within ± 5%.^1^ Deviations from the prescribed dose may worsen the tumor control probability and reduce the overall survival.^2^ If the dose difference is sufficiently significant, the effect can be an unintended harm to the patient or even death. Although radiotherapy is usually delivered as planned, with reported values for the treatment courses with deviations of less than 0.01%,^3^ multiple publications report accidental exposures.^4^, ^5^, ^6^, ^7^ With the ultimate goal of improving patient safety, IAEA safety report 17^7^ details over 90 accidental exposures in radiotherapy. One of the best‐known cases of fatal accidental exposure in radiotherapy is the New York accident.^8^ While saving an intensity‐modulated head and neck treatment plan, an error led to the failure to save the multi‐leaf collimator (MLC) control points. The absence of these control points stored in the record and verify system led to the unintended exposure of the entire neck. The error was only detected through routine quality control (QC) measurements after three treatment fractions had been delivered.

In‐vivo dosimetry has been a safety barrier used in radiotherapy for many years.^9^ In‐vivo dosimetry consists of performing dose measurements during the radiotherapy treatment delivery. The objective is to detect deviations, if any, between the delivered and planned doses, as calculated in the TPS.^9^ Historically, in‐vivo dosimetry has been conducted with point detectors placed on the patient's surface at the beam's entrance and/or exit. However, applying this methodology to modulated techniques involves additional difficulties due to the variation of the detector's response to the dose rate and the uncertainties of point detectors' placement on the patient's skin in high dose‐gradient areas.^10^ Transit dosimetry has overcome the limitations of in‐vivo dosimetry with point detectors.^11^, ^12^, ^13^ Transit dosimetry uses the images obtained with the electronic portal imaging device (EPID) during plan delivery. Transit images are generated by the radiation passing through the patient. Therefore, a change in the attenuation of the treatment beam through the patient will produce a change in the transit image.

Several commercial systems perform automatic transit dosimetry,^11^ one of which is PerFRACTION (Sun Nuclear Corporation, Melbourne, USA). Several users have reported their experience with PerFRACTION.^14^, ^15^, ^16^ Notably, the most comprehensive series of reported experiences is from Bossuyt et al.,^17^ with a 3‐year experience. PerFRACTION converts the transit image to a measured transit dose distribution and compares it with a predicted transit dose distribution. Such predicted transit dose distribution is calculated using the planning CT and the treatment plan with a collapsed‐cone convolution/superposition algorithm. Sánchez‐Artuñedo et al.^18^ also validated the transit dosimetry algorithm with Monte Carlo simulations and ionization chamber measurements.

When a transit image is acquired, PerFRACTION performs a 2D gamma index analysis^19^ to compare the measured and the predicted dose distributions. The users can customize the parameters for the gamma analysis (dose difference, distance to agreement, threshold, and global or local normalization) and the tolerance criteria. Tolerance criteria are established based on the passing rate, the percentage of points with a gamma index value inferior to 1. Thus, prior to its implementation, users need to decide on the gamma criteria to use. As PerFRACTION allows performing gamma index analysis with more than one criterion, the user can apply a standard criterion to track minor deviations and a second criterion to focus on major deviations in the delivered dose that can affect patient safety. This work aims to determine the gamma criterion that maximizes the sensitivity and specificity of PerFRACTION for detecting safety failure modes that potentially introduce large changes in the absorbed dose distribution in external beam radiotherapy.

## 2 MATERIALS AND METHODS

We selected five failure modes reported in the failure modes and effects analysis (FMEA) for a generic intensity‐modulated radiation therapy (IMRT) process reported in the AAPM TG‐100 report.^20^ Failure modes were selected based on their potential to introduce a large deviation in the absorbed dose distribution and their ability to produce a change in the transit dose, which PerFRACTION could potentially detect. The failure modes assessed were: (1) errors related to linac hardware and linac output, (2) errors related to the breathing management protocol, (3) errors in patient identification, (4) use of incorrect CT or wrong adapted plan, and (5) errors in bolus placement.

### 2.1 Erroneous plan selection and calculation

Treatment plans that might potentially present the error were randomly selected for each error type. These plans were considered the base plans. Then, erroneous treatment plans, that is, a set of modified plans containing the introduced errors, were created for each error presented below. Both base and erroneous plans were calculated in Eclipse 15.6 (Varian Medical and Systems) using AcurosXB for a TrueBeam STx 2.7 (Varian Medical and Systems) with an HD120 MLC.

#### 2.1.1 Errors related to linac hardware and linac output

A volumetric modulated arc therapy (VMAT) plan of a head and neck cancer patient with lymph node involvement was selected as the base plan. Treatment prescription was 33 fractions, with a total dose of 54 Gy to the elective nodes (PTV Low) and 70 Gy to the simultaneous integrated boost encompassing both the tumor and the positive nodes (PTV High). The plan was generated using three full arcs with collimator angles set at 286°, 346°, and 46°. Following the SEAFARER methodology,^21^ known errors were intentionally introduced into the base plan using a Python script. The errors included variations in the multileaf collimator (MLC) aperture (± 1.0 and ± 1.5 mm, and +2 and +5 mm exclusively for the central leaves), changes in the collimator angle (± 1°), and in the monitor units (MU) (± 2%, representing output variations), as well as combinations of them. A positive MLC aperture change indicates an increased distance between opposite leaves, while a negative change indicates a reduced gap. A total of 11 erroneous plans were created, 10 following the SEAFARER methodology, with some erroneous plans presenting more than one error per plan, and an eleventh plan simulating the New York incident (Table S1). The planned jaws remained fixed at the same position as the base plan, but the MLC was retracted behind the jaw level. The plan was recalculated using the same MU as the base plan.

#### 2.1.2 Errors related to breathing management

A lung stereotactic body radiotherapy (SBRT) VMAT treatment plan with a prescribed dose of 48 Gy in three fractions to the planning target volume (PTV) was selected as the base plan. The patient was simulated using a 4D‐CT, using a phase‐based binning, dividing the breathing cycle into ten phases. A CT image was reconstructed for each breathing phase, and the gross tumor volume (GTV) was contoured in each CT image. Four CT image sets were created: one selecting the expiration phase (50% of the breathing cycle) CT, one selecting the inspiration phase (0%) CT, an average CT selecting the expiration phases between 30% and 60%, and finally an average CT comprising all the breathing phases. In the expiration and inspiration CTs, the PTV was constructed by adding a 5 mm margin to the GTV. In the 30%–60% CT and the average CT, an internal target volume (ITV) was created as the accumulated GTV of all the phases used. Finally, a 5 mm margin was added to the ITV to create the PTV. Two base plans were created using either the inspiration or expiration CT. Each base plan was recalculated in the other three structure sets, generating a total of six erroneous plans. Each erroneous plan represents a treatment plan delivered with a different breathing management protocol from the planned one.

#### 2.1.3 Errors in patient identification

Two plans from different breast cancer patients (patients A and B) without lymph node involvement were selected as base plans. The treatment prescription was 15 fractions, with a total dose of 40.5 Gy to the breast and 48 Gy to the simultaneous integrated boost. After anonymizing and exporting the treatment plan, each plan was imported to the other patient and recalculated, maintaining the original monitor units and the isocenter position.

#### 2.1.4 Errors due to using an incorrect ct or a wrong adapted plan

A VMAT palliative lung treatment plan with a prescribed dose of 30 Gy in 15 fractions to the PTV was selected as the base plan. After five fractions, the patient was replanned due to a significant decrease in fluid in the ipsilateral lung.

Each plan (the original and the adapted one) was considered the base plan in its corresponding CT. The two erroneous plans were created by recalculating each plan using the alternate CT image while maintaining the MU fixed. These erroneous plans represent either a treatment planned utilizing an incorrect CT structure set (such as the use of a previous treatment CT or an invalid CT) or an erroneous selection of the plan to be delivered in an adaptive radiotherapy workflow.

#### 2.1.5 Errors in bolus placement

The base plan was an IMRT plan for a palliative breast treatment of an ulcerating tumor, with a prescribed dose of 24 Gy in 3 fractions to the PTV. Both the GTV and the clinical target volume (CTV) encompassed the patient's skin. A 1 cm‐thick virtual bolus was added in Eclipse with mass density set to 1 g/cm^3^, that is, Hounsfield Units set to zero, to ensure sufficient coverage of the CTV in the absence of bolus during treatment. The erroneous plan was created and recalculated without the bolus while maintaining the same fluences and MU as the base plan.

### 2.2 Data collection and analysis

Dosimetric parameters for the PTVs and OARs were extracted from base plans and erroneous plans from Eclipse using visual scripting. The change in a dosimetric parameter when a failure mode is present was calculated as the difference between the value in the erroneous plans and the value in the base plan. Table 1 presents the changes in the dosimetric parameters calculated for the erroneous plans with respect to the corresponding base plan for each failure mode.

**TABLE 1:** Changes in the dosimetric parameters calculated for the erroneous plans with respect to the corresponding base plan for each failure mode.

Failure mode	OAR	OAR dosimetric parameter	PTVs dosimetric parameters
I) Linac hardware and linac output	Spinal cord	Δ*D*max	ΔDmean, ΔDmax, Δ*D*95%, Δ*D*98%, Δ*D*2%
	Parotids	Δ*D*mean	
	Pharynx		
	Larynx		
II) Breathing management protocol	Ipsilateral lung		
III) Patient identification	Heart, ipsilateral lung		
IV) Incorrect CT or wrong adapted plan			
V) Bolus placement			

Once base plans and erroneous plans from the five failure modes presented were created, the plans were imported to PerFRACTION (V. 2.10.0). The import of the DICOM files is the mandatory first step in the transit dosimetry workflow, so the platform can calculate the expected transit dose distribution. Every time the treatment plan is delivered in the treatment unit, where a transit image is acquired, PerFRACTION will compare the expected and the measured transit dose distributions. Therefore, all plans were delivered, in air, in a TrueBeam STx 2.7 with the EPID extended at a source‐imager distance of 150 cm to trigger a fraction analysis.

Once the analysis of the treatment fraction was completed, expected and measured transit dose distributions could be exported from the platform. Only expected transit dose distributions from base plans and erroneous plans were exported from the platform. The expected transit dose of an erroneous plan is equivalent to the measured transit image obtained after the delivery of the base plan when the error represented in the erroneous plan occurs. We compared the expected transit images of the erroneous plans with their corresponding base plans using the SNC Patient Software V.8.8.1 (Sun Nuclear Corporation, Melbourne, USA). The 2D gamma criteria studied were: γ(3%/3 mm), γ(5%/3 mm), γ(5%/5 mm), γ(7%/5 mm), γ(10%/1 mm), γ(10%/3 mm), and γ(10%/5 mm). All the gamma criteria were evaluated with local and global normalization, leading to a total of 14 gamma metrics evaluated. The dose threshold was set at 20%, and the tolerances were set at a 95% passing rate in all cases. The gamma criteria analyzed were based on the empirically determined parameter for gamma analysis by Bossuyt et al.^17^

We assessed the sensitivity and specificity of each gamma criterion by categorizing the 22 erroneous plans based on the absolute PTV dosimetric parameter variation greater than 5%, 10 %, and 20%, for each of the dosimetric parameters presented in Table 1. In the treatment plans with different PTV dose levels, the criteria were applied to the higher‐dose PTV. Each failure mode was classified as positive (1) or negative (0) based on whether the dosimetric variation exceeded the condition under study. For example, if the condition studied was Δ*D*mean > 10% and a plan had a Δ*D*mean = 6.3% it was classified as 0, whereas a plan with a Δ*D*mean = 13.3% was classified as 1.

A receiver‐operating characteristic (ROC) curve characterizes the performance of a binary classifier across a range of threshold values, represented in our study by the 2D gamma passing rates. Each of the passing rates obtained under a fixed gamma criterion was set as the decision threshold. Failure modes with passing rates above the threshold were classified as negative, while those below were classified as positive. For each threshold, sensitivity and specificity were calculated. Sensitivity, or true positive rate (TPR), measures the proportion of the positive responses that are correctly identified as positive by the classifier. Specificity measures the proportion of the negative responses that are correctly identified as negative by the classifier. The ROC curve plots sensitivity against 1‐specificity, or false positive rate (FPR). The shape of the ROC curve reflects predictive performance: curves closer to the top‐left corner indicate better classification, while the diagonal represents a random classifier. The area under the curve (AUC) of a ROC curve summarizes the classifier's overall discriminative ability.^22^ The optimal cut‐off point corresponds to the passing rate that maximizes the sensitivity while minimizing the false positive rate. Mathematically, it is expressed as the maximum of the Youden's J statistic (max(sensitivity + specificity‐1)).

ROC curves were generated using RStudio (V.2023.09.0+463) with R (V.4.2.2) using the pROC package.^23^ For each ROC curve, both the AUC and the optimal threshold were calculated.

The AUC of a ROC curve evaluates the performance of a test to classify between positive or negative, or, in the context of the present study, if a failure mode that produces a difference in a dosimetric parameter > x % is detected or not. According to Mandrekar et al.,^24^ if the AUC value of a test to classify positive or negative is 0.8 to 0.9, the test is excellent, and above 0.9, the test is outstanding.

## 3 RESULTS

Below, we show the changes in the dosimetric parameters and the comparison of the transit images for each of the five failure modes studied.

### 3.1 Errors related to Linac hardware and Linac output

Table S1 summarizes the changes in the PTV mean dose and OAR dosimetric parameters of the head and neck erroneous plans with respect to the base plans. Similar results were obtained for the rest of the PTV dosimetric parameters studied.

**FIGURE 1:**
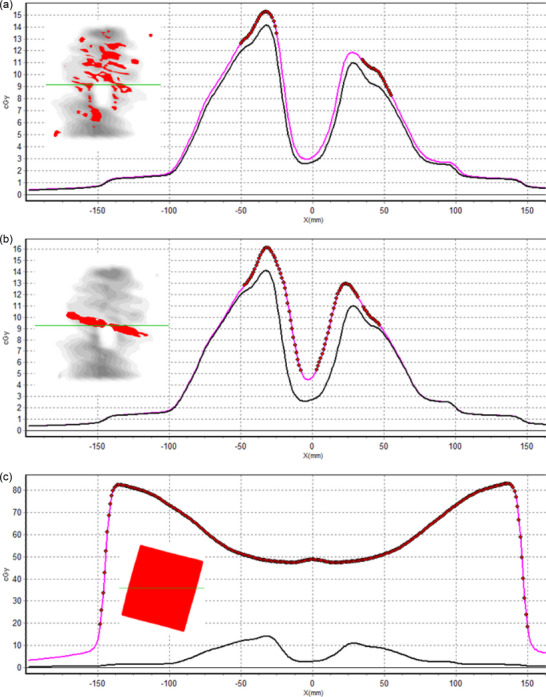
Errors related to linac hardware and linac output. (a) Gamma comparison of an erroneous plan with each leave retracted one mm respect base plan; (b) Only central leaves are retracted 5 mm respect base plan; (c) Represents the New York incident in which all the MLC leaves were retracted behind the position of the jaws. The gamma comparison of both plans is overprinted at the left comparison with a global γ(10%/1 mm) of the dose profiles. In grey, the areas have a gamma value of less than 1; in red, the areas have a gamma value higher than 1, and the dose of the erroneous plan is higher than the base plan. The green line indicates the position of the plotted profile. The black line represents the dose profile of the base plans, and the pink line is the dose profile of the erroneous. The red dots represent points with a gamma value higher than one and a dose of the erroneous plan higher/lower than the base plan.

Figure 1 presents a profile and the gamma index comparison between the transit doses of three of the head and neck erroneous plans and the base plans. Figure 1a shows the gamma comparison of an erroneous plan with all leaves retracted 1 mm with respect to the base plan. In Figure 1b, only the central leaves were retracted 5 mm with respect to the base plan. Finally, Figure 1c simulates the New York incident in which all the MLC leaves are retracted behind the position of the jaws. In cases Figure 1a,b, the change in MLC position is translated into a change of the field penumbra and an increase in the delivered dose. In Figure 1c, the absence of the MLC increased the delivered absorbed dose due to the higher ratio of Dose/MU of VMAT with respect to three‐dimensional conformal radiation therapy (3D‐CRT) techniques. Table S2 presents the passing rates obtained in all the failure modes studied for each of the gamma criteria analyzed.

**FIGURE 2:**
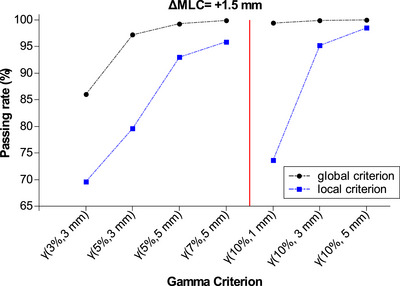
Gamma passing rate for different gamma criteria for the erroneous plan with an introduced error in the MLC aperture of + 1.5 mm. Data points are connected for visual aid. The red vertical line distinguishes between the below 10% and the 10% dose difference criteria.

Figure 2 shows the passing rates obtained for the different gamma criteria evaluated for the erroneous plan with an introduced error in the MLC aperture of + 1.5 mm. The most significant difference between the two criteria is obtained with criterion γ(10%/1 mm), with a passing rate of 99.4% obtained with global normalization and 73.6% with local normalization.

**FIGURE 3:**
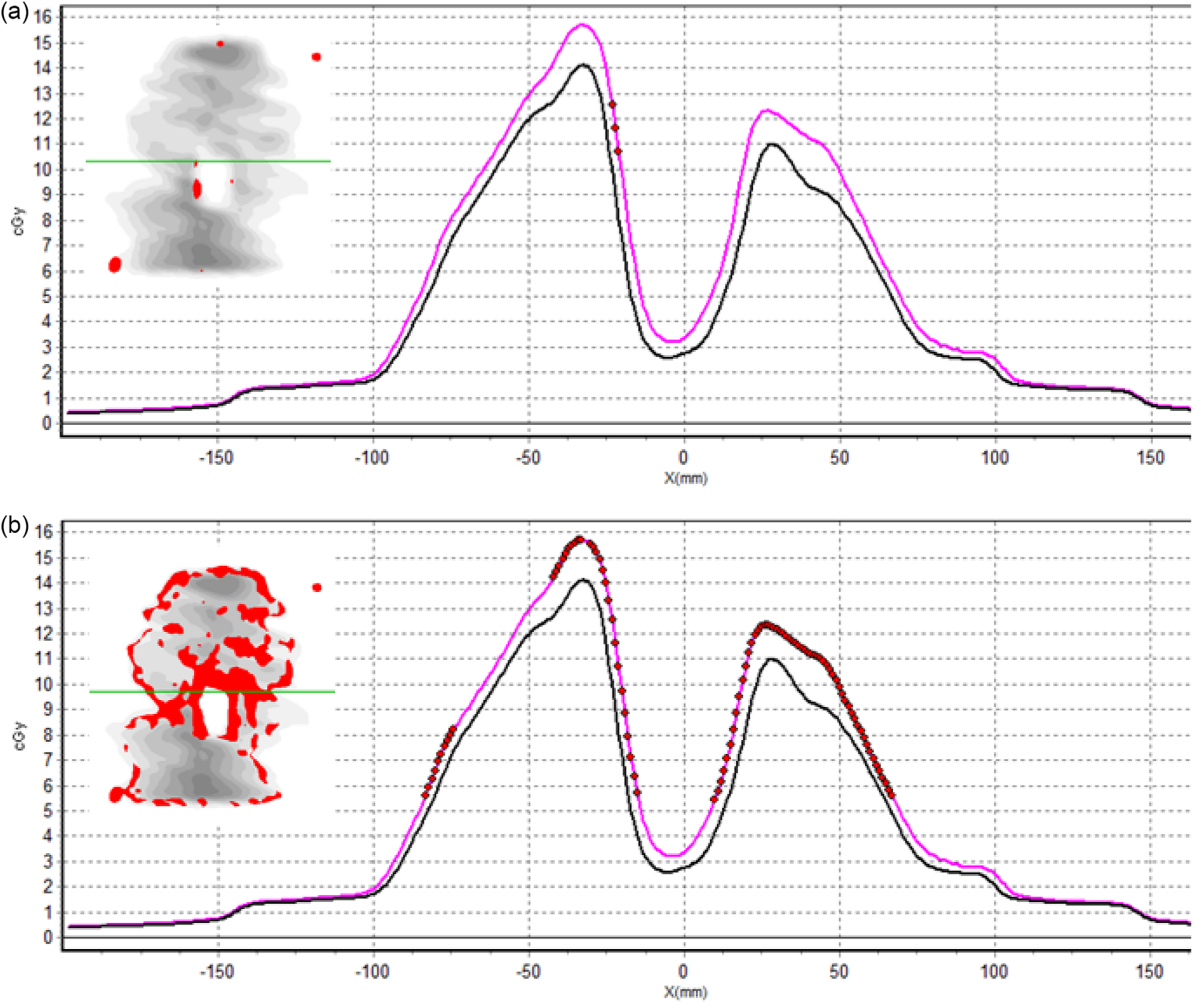
Gamma index comparison of the erroneous plan with an introduced error in the MLC aperture of +1.5 mm. (a) Comparison with a global γ(10%/1 mm) and (b) comparison with a local γ(10%/1 mm). The gamma comparison of both plans is overprinted on the left side of the dose profiles. In grey, the areas have a gamma value of less than 1; in red, the areas have a gamma value higher than 1, and the dose of the erroneous plan is higher than the base plan. The green line indicates the profile represented graphically. The black line represents the dose profile of the base plans, and the pink line is the dose profile of the erroneous. The red dots represent points with a gamma value higher than one and a dose of the erroneous plan higher than the base plan.

Figure 3 presents the gamma index comparison of the erroneous plan with an introduced error in the MLC aperture of + 1.5 mm. Figure 3a presents the comparison with a global γ(10%/1 mm) and Figure 3b with a local γ(10%/1 mm). A more restrictive criterion, such as local gamma comparison, highlights the differences in the field penumbra and the increase in the delivered dose. Therefore, local gamma criteria are more sensitive to detecting mechanical or output‐related failure modes.

### 3.2 Errors related to breathing management

Table S3 summarizes the changes in the PTV parameters of the lung erroneous plans, which were created to represent errors related to the breathing management protocol compared to the base plans. The average change in the lung mean dose was −0.4% and −0.2% in the V20Gy(%).

**FIGURE 4:**
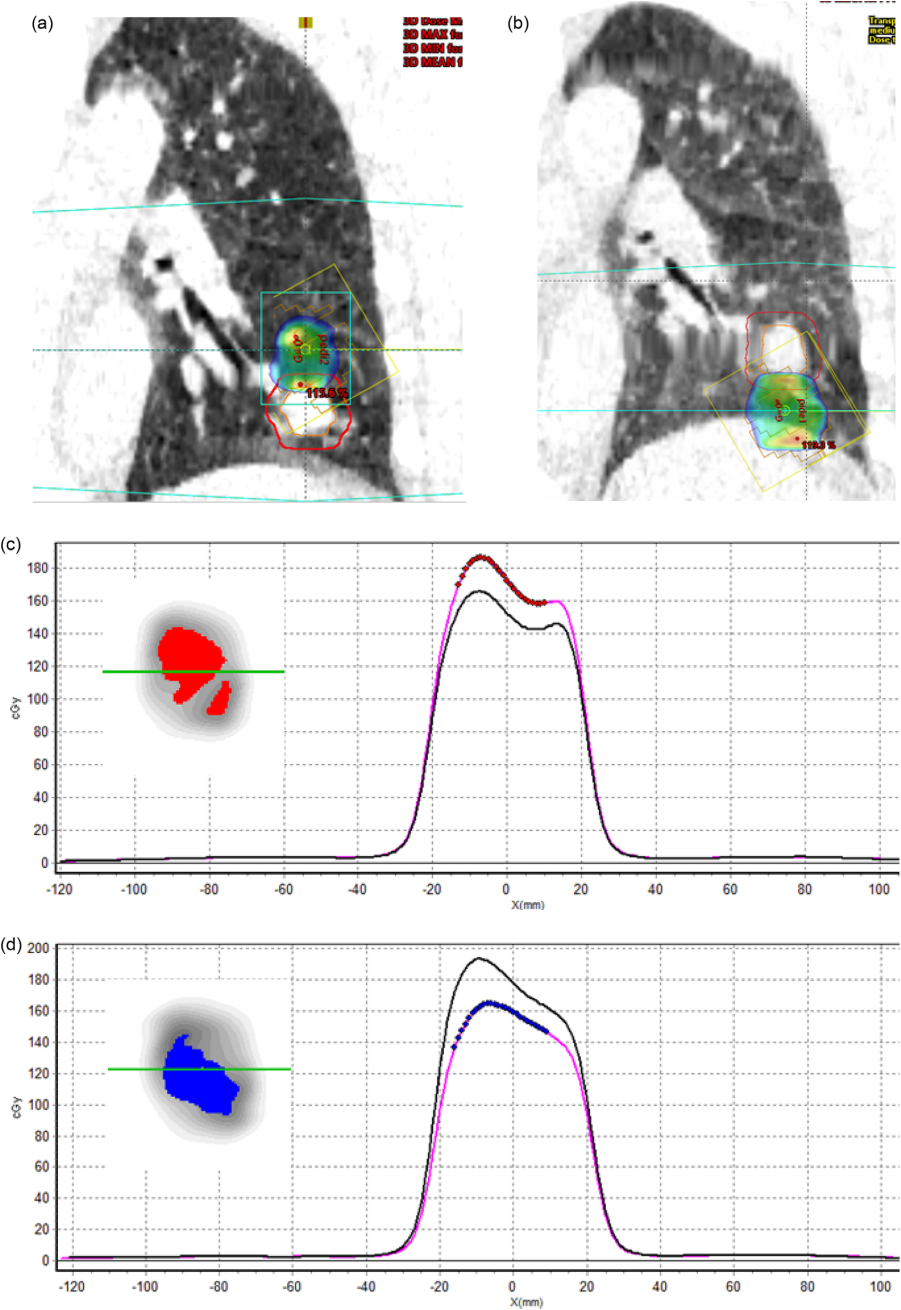
Errors related to breathing management. (a) and (b) present the dose distribution of the plans delivered in inspiration and expiration while planned conversely. GTV is presented as an orange contour and PTV in red. (c) Presents transit dosimetry of the plan delivered in inspiration while planned in expiration (Figure 4a), and (d) delivered in expiration and planned in inspiration (Figure 4b); the gamma comparison of both plans is overprinted on the left side of the dose profiles. In grey, the areas have a gamma value of less than 1; in red/blue, the areas have a gamma value higher than 1, and the dose of the erroneous plan is higher/lower than the base plan. The green line indicates the profile represented graphically. The black line represents the dose profile of the base plans, and the pink line is the dose profile of the erroneous. The red/blue dots represent points with a gamma value higher than one and a dose of the erroneous plan higher/lower than the base plan.

As expected, the most notable differences were obtained when delivering the treatment in expiration/inspiration, when the treatment was planned with the inspiration/expiration CT images. Figure 4a,b show the transit dose distribution of the plans delivered in inspiration and expiration, while planned conversely. The shift in the breathing phase produced a PTV miss in both cases. Figure 4c presents the results obtained with the plan delivered in inspiration while planned in expiration. As the lung has a density lower than the GTV, the beam is less attenuated, so the dose in the transit image is higher than in the base plan. Conversely, when treated in expiration, soft tissue attenuates part of the beam instead of lung tissue, leading to a lower dose than expected in the transit image, as presented in Figure 4d.

The passing rates obtained when delivering the plan in expiration, while planned in inspiration, failed the tolerance established independently of the gamma criteria set (Table S2).

### 3.3 Errors in patient identification

Treating patient A using the treatment plan of patient B produced a change in the boost PTV coverage, Δ*D*95% of −91.8% and of −70.4% for the breast PTV. The mean change in the lungs mean dose was −5% and −6.5% in the V20Gy(%). Conversely, when treating patient B with the treatment plan of patient A, the change in PTV coverage was −15.5% and −9.4% for the boost and the breast, respectively, and the lung mean dose and the V20Gy(%) increased by + 7% and + 8.8%, respectively. In both scenarios, the heart mean dose increased by + 0.3%. The passing rate failed the tolerance established independently of the gamma criteria set (Table S2).

**FIGURE 5:**
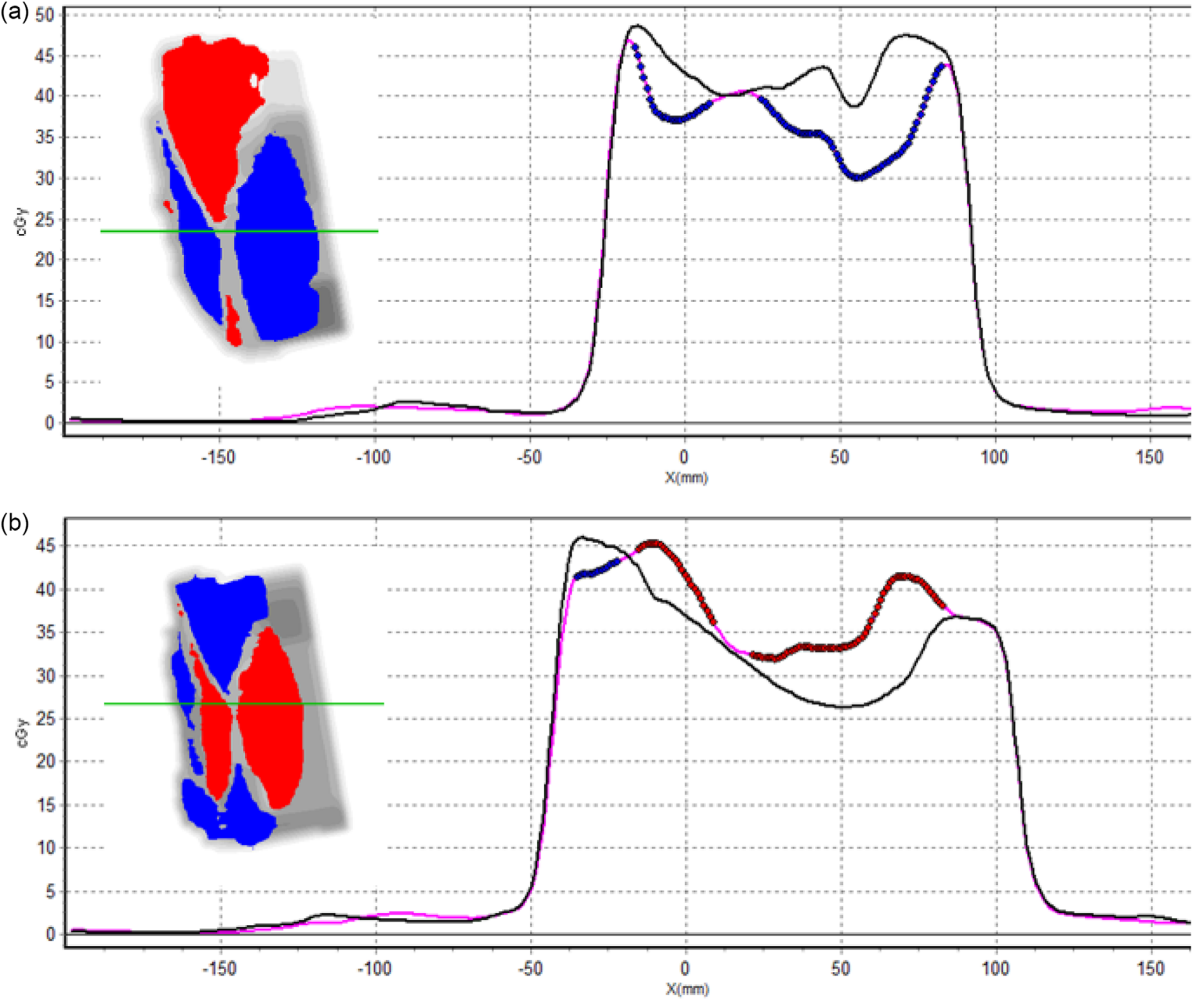
Errors in patient identification. (a) Presents the transit dosimetry of patient A treated with a plan of patient B, and (b) conversely. The gamma comparison of both plans is overprinted on the left side of the dose profiles. In grey, the areas have a gamma value of less than 1; in red/blue, the areas have a gamma value higher than 1, and the dose of the erroneous plan is higher/lower than the base plan. The green line indicates the profile represented graphically. The black line represents the dose profile of the base plans, and the pink line is the dose profile of the erroneous. The red/blue dots represent points with a gamma value higher than one and a dose of the erroneous plan higher/lower than the base plan.

Figure 5a,b presents the transit dosimetry of patient A treated with a plan of patient B conversely.

### 3.4 Errors using incorrect ct or wrong adapted plan

**FIGURE 6:**
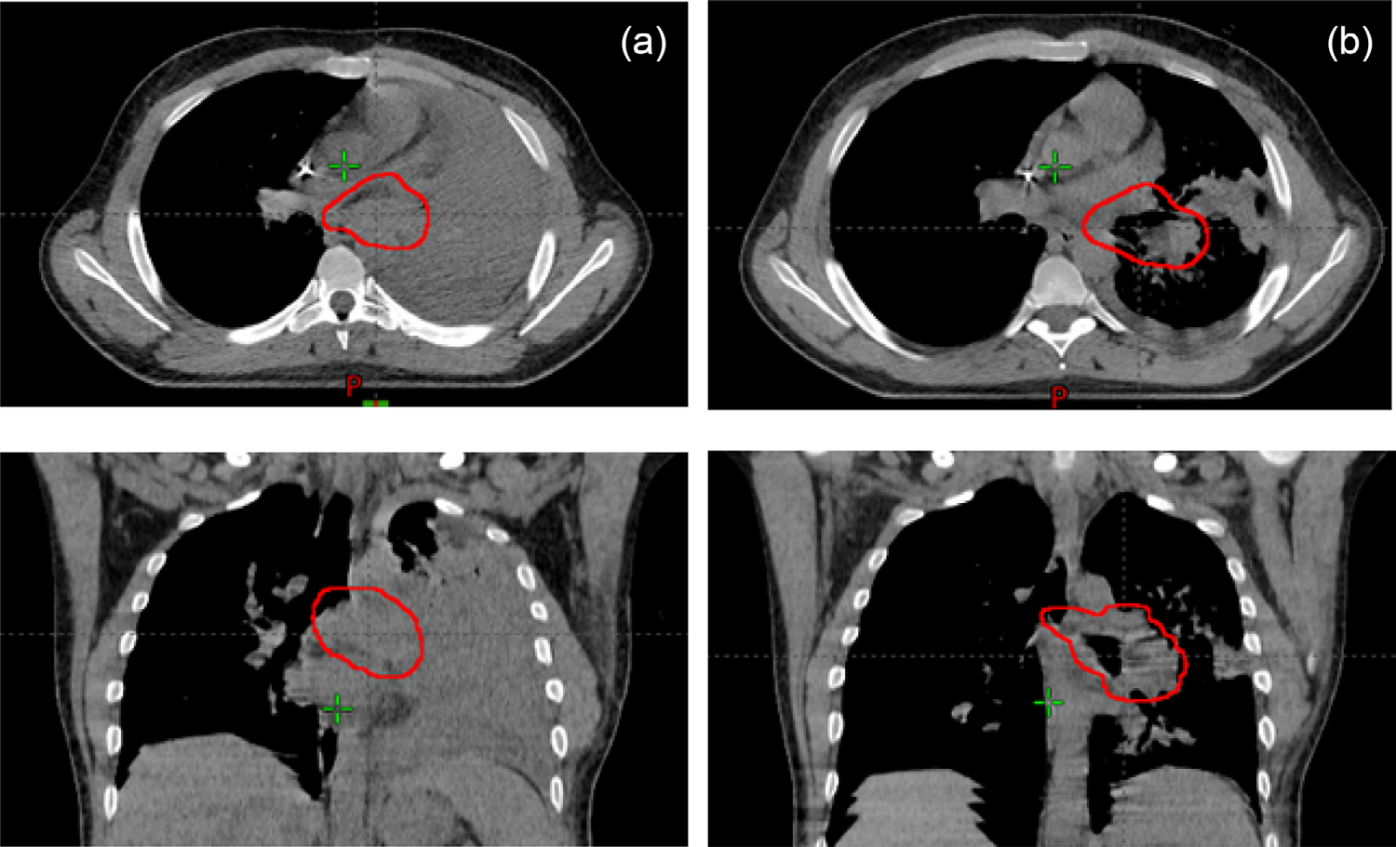
(a) Initial planning CT with the PTV contoured in red and (b) planning CT after five treatment fractions.

Figure 6 shows the planning CT at the beginning of treatment and the replanned CT. Treating a patient with the anatomy corresponding to the original CT but using the plan calculated with anatomical changes produced a change in the PTV coverage Δ*D*95% of −72.8%, an increase of lung mean dose and heart mean dose of 0.2%, and a 5%, respectively. A difference of −91.3% in PTV coverage and a decrease in lung mean dose and heart mean dose of −0.4% and 5.8% were obtained in the opposite combination of delivered and planned. The difference in attenuation between lung and water/soft tissue leads to an under‐ or overdosage of transit dosimetry. The passing rate failed the tolerance established independently of the gamma criteria set (Table S2).

### 3.5 Errors in bolus placement

Treating a patient without using the 1 cm bolus as planned produced a change in the coverage Δ*D*95% of −39.7% for the PTV and −21.9% of the GTV. The absence of the attenuation produced by the bolus led to an overdosage of the transit image. In 8 out of the 14 gamma criteria evaluated, three global criteria, and five local criteria, the passing rate failed the tolerance established (Table S2).

### 3.6 Analysis results

**FIGURE 7:**
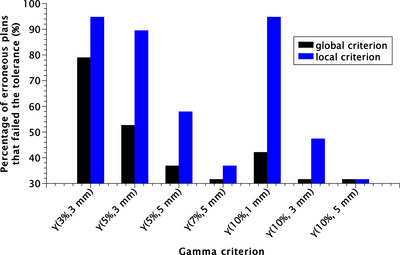
The percentage of erroneous plans failing the tolerance passing rate was established. The black columns represent the global gamma criteria, and the blue columns represent the local gamma criteria.

Figure 7 presents the percentage of erroneous plans failing the established tolerance passing rate, depending on the criterion set to perform the gamma analysis. The gamma criteria with the most erroneous plans failing the established tolerance were local γ(3%/3 mm) and γ(10%/1 mm); the most sensitive global criterion was γ(3%/3 mm).

**FIGURE 8:**
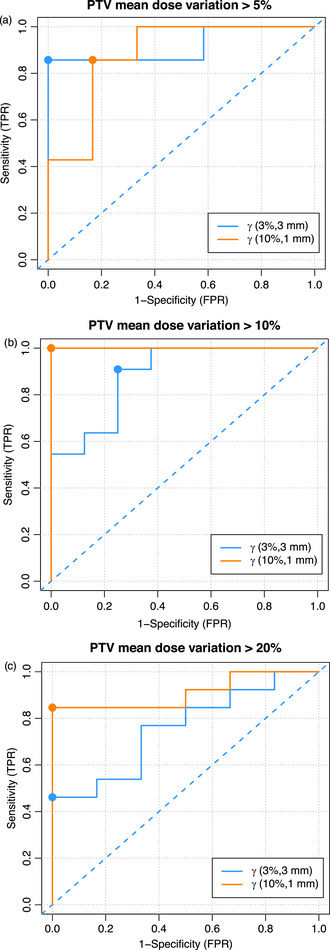
ROC curves obtained using global γ(3%/3 mm) and γ(10%/1 mm) criteria. The *x*‐axis represents the false positive ratio (FPR) or 1‐specificity, and the *y*‐axis represents the true positive ratio (TPR) or specificity. The dashed line represents the line of no‐discrimination. The point in each ROC curve represents the maximum value of Youden's J statistic from which the optimal cut‐point passing rate is determined. (a) Failure modes that produce a change in the PTV mean dose above 5%, (b) above 10%, and (c) above 20%.

The sensitivity and specificity of the fourteen gamma criteria were assessed regarding the change in the PTV mean dose produced for each erroneous plan described above. Figure 8 presents the ROC curves obtained using global γ(3%/3 mm) and γ(10%/1 mm) criteria to assess the performance of PerFRACTION to detect failure modes that produce a change in the PTV mean dose above 5% (Figure 8a), 10% (Figure 8b), or 20% (Figure 8c). Tables 2 and 3 present the AUC of the ROC curves obtained using different global and local gamma criteria, respectively, and the difference in PTV Δ*D*mean (%).

**TABLE 2:** AUC of the ROC curves and optimal passing rate with different global gamma criteria. ROC curves were created regarding the change in the PTV mean dose produced for each erroneous plan and each global gamma criterion.

	PTV Δ*D*mean (%) > 5%		PTV Δ*D*mean (%) > 10%		PTV Δ*D*mean (%) > 20%	
Gamma criteria	AUC	Optimal passing rate (%)	AUC	Optimal passing rate (%)	AUC	Optimal passing rate (%)
γ(3%/3 mm)	0.92	91.4	0.89	77.8	0.76	91.4
γ(5%/3 mm)	0.92	96.3	0.97	95.3	0.85	95.3
γ(5%/5 mm)	0.80	88.3	0.88	88.3	0.74	88.3
γ(7%/5 mm)	0.74	92.6	0.85	92.6	0.71	92.6
γ(10%/1 mm)	0.88	98.4	1.00	96.4	0.91	96.4
γ(10%/3 mm)	0.85	98.9	0.93	97.2	0.83	97.2
γ(10%/5 mm)	0.76	99.0	0.85	96.7	0.72	96.7

**TABLE 3:** AUC of the ROC curves and optimal passing rate with different local gamma criteria. ROC curves were created regarding the change in the PTV mean dose produced for each erroneous plan and each local gamma criterion.

Gamma criteria	PTV Δ*D*mean (%) > 5%		PTV Δ*D*mean (%) > 10%		PTV Δ*D*mean (%) > 20%	
	AUC	Optimal passing rate (%)	AUC	Optimal passing rate (%)	AUC	Optimal passing rate (%)
γ(3%/3 mm)	0.84	79.3	0.76	50.4	0.62	50.4
γ(5%/3 mm)	0.89	88.4	0.83	68.2	0.70	68.2
γ(5%/5 mm)	0.83	96	0.81	90.6	0.67	90.4
γ(7%/5 mm)	0.83	98.1	0.80	90.4	0.67	90.6
γ(10%/1 mm)	0.97	82.5	0.94	73.6	0.81	73.6
γ(10%/3 mm)	0.95	95.7	0.91	94.2	0.79	94.2
γ(10%/5 mm)	0.76	97.1	0.80	97.1	0.66	97.1

The mean AUC for global gamma criteria was 0.84, 0.91, and 0.79 for erroneous plans that produced a change in the PTV mean dose > 5%, > 10%, and > 20%, respectively. When using local gamma criteria, the mean AUC was 0.87, 0.83, and 0.70, respectively. With global normalization, all the AUC values calculated were above 0.7. Still, with local criteria, only the AUC calculated using a γ(10%/1 mm) or a γ(10%/3 mm) to detect errors that produce a difference in PTV mean dose > 20% were above 0.7.

For errors that produce a difference in PTV mean dose greater than 5%, an AUC above 0.9 was obtained using global γ(3%/3 mm), γ(5%/3 mm), and local γ(10%/1 mm) and γ(10%/3 mm) normalizations. For PTV mean dose differences above 10%, AUC values above 0.9 were obtained using global γ(5%/3 mm), γ(10%/1 mm), γ(10%/3 mm), and local γ(10%/1 mm) and γ(10%/3 mm). The only gamma criterion with an AUC value above 0.9 was global γ(10%/1 mm) when evaluating the performance of transit dosimetry to detect deviations in the PTV mean dose above 20%. Similar results were obtained for the rest of the PTV parameters analyzed.

**FIGURE 9:**
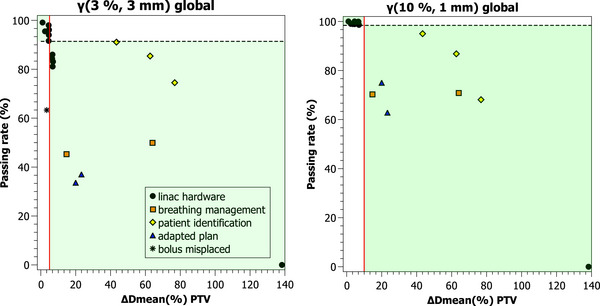
Passing rates obtained using global (a) γ(3%/3 mm) and (b) γ(10%/1 mm) criterion for the erroneous planes studied. The dashed line represents the optimal passing rate obtained from ROC analysis. The red line represents a 5% mean dose difference in the PTV in (a) and (b) a 10%. The legend is presented only in (a) for simplicity.

Using a more restrictive gamma criterion would lead to a more considerable decrease in the passing rate at a fixed dose difference. Thus, establishing the tolerance level with the optimal passing rate calculated increases the sensitivity and specificity of PerFRACTION. Figure 9a shows the passing rates obtained using a global γ(3%/3 mm) criterion. A vertical red line represents a change in the PTV mean dose above 5%, and the dashed horizontal line is the optimal passing rate obtained from the ROC analysis. The green areas represent either an erroneous plan producing a change in the PTV mean dose above 5% and a passing rate below the optimal passing rate, or a change in the PTV below 5% and a passing rate above the tolerance. Figure 9b presents the passing rate obtained using a global γ(10%/1 mm), and the vertical red line represents a change in the PTV mean dose above 10%.

## 4 DISCUSSION

Based on the gamma metrics evaluated, a global gamma criterion γ(10%/1 mm) optimizes PerFRACTION sensitivity and specificity to detect safety failure modes that produce a change in the PTV mean dose above 10% (Figure 9b). A more standard global gamma criterion γ(3%/3 mm) or γ(5%/3 mm) maximizes PerFRACTION™ sensitivity and specificity to detect deviations in the PTV mean dose above 5%. In both cases, the AUC obtained was above 0.9, implying that PerFRACTION is a test^24^ that can positively detect deviations in the delivered dose. Since PerFRACTION allows performing gamma index analysis with more than one criterion, using two criteria in the analysis could be a balanced approach. Users could apply a standard criterion, such as global γ(3%/3 mm), to track minor changes in the transit image and a global γ(10%/1 mm) criterion with a high passing rate tolerance of 96%–97% to detect errors related to patient safety.

In the analyzed failure modes related to Linac hardware, excluding the erroneous plan representing the New York incident, the change in PTV mean dose was below 7%. The primary contributor to the absorbed dose difference in the erroneous plans with respect to the base plans was the differences in the MLC positioning. According to Enomoto et al.,^25^ the dosimetric impact of random positional errors of MLC positioning of 1 mm is approximately 0.2 % in the generalized equivalent uniform dose (gEUD). In comparison, a systematic error of 1 mm will introduce a dose difference between 2%–9%, depending on the treatment site and the TPS. Therefore, absorbed dose differences greater than 5% are unlikely to occur solely due to random mechanical variations in MLC positioning. However, in plans with high modulation,^26^, ^27^, ^28^, ^29^, ^30^ MLC positioning differences may adversely affect plan quality.

For linac hardware errors, the global γ(10%/1 mm) criterion yielded passing rates above 97.9%, except for the fully retracted MLC failure mode (Table S2). Conversely, using the local γ(10%/1 mm) criterion, all plans failed the tolerance, except for the erroneous plan with a 2 mm aperture modification of the central leaves. Figure 3b shows that a local gamma criterion is more sensitive to differences in the field penumbra and the increase in the delivered dose when an MLC aperture error of + 1.5 mm is introduced. For the same erroneous plan, Figure 2 shows that local normalization results in lower passing rates than global normalization. Therefore, local normalization can enhance the detectability of mechanical or output‐related failure modes. While measurement‐based QC of treatment plans^31^ can detect discrepancies in MLC positioning, independent dose calculation software has been reported to perform better^32^ in detecting suboptimal plans. As PerFRACTION calculates the dose distribution using its independent algorithm, it can also detect failures related to poor commissioning of the TPS or the MLC.

We presented a lung patient plan simulated during the inspiration phase and treated during the expiration phase, and vice versa, as an example of an error associated with the breathing management protocol. Between these two extreme scenarios, numerous minor errors can arise, such as incorrectly setting the gating threshold or a lack of reproducibility of the respiratory curve acquired during simulation. All these errors can lead to an underdosing of the lesion, partial or total (target miss), and can result in an unwanted dose deposition on healthy tissue. Besides the ipsilateral lung, depending on the position of the PTV, other OARs could be affected: the brachial plexus if the PTV is in the lung apex, esophagus, trachea, or bronchial tree if it is a central or ultra‐central lesion, and the stomach or the liver if the PTV is located in the base of the lungs. However, when the treatment was planned in expiration but delivered either in the 30–60 breathing phases or in free‐breathing, PerFRACTION was unable to detect the deviation. Transit dosimetry, and hence PerFRACTION, can only detect deviations that produce differences in the beam attenuation.

In the case of patient misidentification, a breast treatment was considered. Misidentification errors are more likely to occur between treatments of the same anatomical site. Breast cancer has the highest prevalence in the female population,^33^ and therefore, this failure mode could have a moderate probability of occurrence. The severity of this error could be aggravated if the swapped treatments have different doses per fraction, for example, 2 Gy versus 5 Gy, if they involve irradiation of different nodal levels, or differ in laterality. However, the greater the difference between the two treatments, the more likely it is that other safety barriers involved in the radiotherapy process, such as source‐surface distance, image guidance, or the patient themself, will detect the error.

The ROC analysis showed that the sensitivity of a gamma criterion to detect dosimetric deviations varied depending on the magnitude of the dose difference. As shown in Table 2, 3, 3%/3 mm gamma criteria achieved superior discriminative performance for detecting failure modes that produce a change in the PTV mean dose above 5%. For failure modes that produce a larger change in the PTV mean dose, the test becomes less selective. Conversely, 10%/1 mm achieved superior discriminative performance for failure modes that produce changes above 10%. The ROC curves (Figure 8a–c) illustrate these trends, with curves shifting closer to the top‐left corner as sensitivity improves. Moreover, the ROC analysis presented for transit dosimetry can be applied to other safety barriers.

Figure 9a shows that the linac‐related errors studied—except the New York incident—lead to a lower impact on patient safety compared to errors in patient identification or respiratory management protocol. Nevertheless, most QC barriers are focused on the technical treatment delivery phase. Of the five failure modes studied, only the first one, the errors related to linac hardware or the linac output, can be detected with a standard QC pretreatment program^31^ if the failure is presented prior to the treatment plan verification. Also, the rest of the failure modes cannot be detected with an independent dose calculation software based on treatment logs. Therefore, transit dosimetry complements a QC program that monitors the treatment delivery throughout the whole treatment. We focused on transit dosimetry, and while interesting, the analysis of the rest of the PerFRACTION modules is outside the scope of this work.

Figure 7 shows that a higher percentage of erroneous plans failed the tolerance when using a local gamma criterion compared to a global criterion. Although stricter gamma criteria can increase sensitivity, they may also lead to a higher false positive rate, depending on the dose difference evaluated. As shown in Tables 2 and 3, using global gamma normalization, higher AUC values were obtained for failure modes introducing a change in the PTV mean dose exceeding 10% or 20%, reflecting better overall classification performance. In clinical practice, false positives lead to unnecessary resource consumption—time, personnel, and cost—while false negatives compromise treatment efficacy and patient safety. ROC‐based evaluation can therefore optimize decision thresholds in clinical workflows, balancing operational efficiency and patient safety.

The five failure modes studied are not actual incidents presented at any institution, although they may occur in a radiotherapy department. Large failure modes were selected based on whether they can produce a change in the transit dose and, therefore, be potentially detected by PerFRACTION. Transit dosimetry will only detect errors that produce a difference in the beam attenuation with respect to the predicted transit images. Hence, errors related to patient data, such as staging or medical data crossed between two patients, ignoring previous treatments, incorrect contouring, missed positive nodes, or prescription errors, cannot be detected. Future work would be to reproduce the failure modes in a phantom to validate the optimal gamma criterion determined. An FMEA analysis that generates risk priority numbers for the five failure modes in this work would make an excellent companion study. Moreover, we considered whether an independent dose calculation pretreatment quality assurance software could detect the failure modes presented, but this was deferred to future work.

We studied the sensitivity of PerFRACTION under errors that can affect patient safety, with absorbed dose differences in the PTV exceeding 20%. To our knowledge, this is the first work studying the optimal gamma criteria and tolerance passing rate to enhance PerFRACTION sensitivity and specificity.

## 5 CONCLUSION

A global γ(10%/1 mm) criterion maximizes the sensitivity and specificity of PerFRACTION to detect erroneous deliveries at a level of dose differences of 10% or greater in the PTV. For failure modes that produce differences below 10% the optimal gamma criterion was found to be global γ(3%/3 mm).

## AUTHOR CONTRIBUTIONS

All authors contributed to the conception of the study, the data analysis, and the manuscript writing.

## CONFLICT OF INTEREST STATEMENT

The authors declare no conflicts of interest.

## Supporting information



Supporting Information

Supporting Information

Supporting Information
