# Supplementary material for: Determining optimal transit dosimetry gamma parameter values for the detection of failure modes using receiver operating curve analysis
Source: J Appl Clin Med Phys. 2025 Dec 29;27(1):e70424. doi: 10.1002/acm2.70424 (PMC12746048; doi:10.1002/acm2.70424)
Supplement: Supplementary file 1 — Supporting Information [file ACM2-27-e70424-s003.docx]

Supplemental Table 1. Changes in PTV and OAR dosimetric parameters of the linac hardware and linac output head and neck erroneous plans with respect to base plans. The first three columns show the parameter modified to represent failure modes related to Linac hardware and Linac output. As expected, a positive change in the MLC or the monitor units implies an increase in the absorbed dose in the PTVs. Conversely, decreasing the leaves gap or the monitor units reduces the PTV mean absorbed dose.

| Induced failure mode: Linac hardware | | | PTV High | PTV Low | Right parotid | Left parotid | Larynx | Pharynx | Spinal canal |
| --- | --- | --- | --- | --- | --- | --- | --- | --- | --- |
| ΔMLC (mm) | ΔCollimator angle (°) | ΔMU | ΔDmean (%) | ΔDmean (%) | ΔDmean (%) | ΔDmean (%) | ΔDmean (%) | ΔDmean (%) | ΔDmax (Gy) |
| -1.5 | - | - | -6.7 | -4.6 | -4.1 | -4.2 | -5 | -5.6 | -5.5 |
| -1.0 |  |  | -4.5 | -3.1 | -2.8 | -2.9 | -3.5 | -3.9 | -3.8 |
| -1.0 | -1.0 |  | -4.5 | -3.1 | -3.1 | -3.8 | -3.5 | -3.7 | -3.2 |
| -1.0 | -1.0 | -2.0% | -6.5 | -4.7 | -3.7 | -4.4 | -4.3 | -4.4 | -3.8 |
| +1.5 |  |  | 7 | 4.6 | 5.1 | 5.8 | 5.8 | 7.2 | 6.7 |
| +1.0 |  |  | 4.7 | 3.1 | 3.3 | 3.8 | 3.8 | 4.7 | 4.4 |
| +1.0 | +1.0 |  | 4.6 | 3 | 4.1 | 5.2 | 3.9 | 4.6 | 4.3 |
| +1.0 | +1.0 | +2.0% | 6.8 | 4.7 | 4.9 | 6.0 | 4.8 | 5.4 | 5.2 |
| +5 (central leaves) |  |  | 2.4 | 0.9 | 0.4 | 0.2 | 3.7 | 4.5 | 5.5 |
| +2 (central leaves) |  |  | 1 | 0.3 | 0.1 | 0.1 | 1.4 | 1.7 | 1.4 |
| Fully retracted | - | - | 138.3 | 157.5 | 195 | 202.2 | 205.1 | 206.6 | 137 |
