# Supplementary material for: Determining optimal transit dosimetry gamma parameter values for the detection of failure modes using receiver operating curve analysis
Source: J Appl Clin Med Phys. 2025 Dec 29;27(1):e70424. doi: 10.1002/acm2.70424 (PMC12746048; doi:10.1002/acm2.70424)
Supplement: Supplementary file 2 — Supporting Information [file ACM2-27-e70424-s002.docx]

Supplemental Table 3. Changes in the PTV dosimetric parameters of the incorrect breathing management protocol lung erroneous plans with respect to the base plans. Erroneous plans were created to represent failure modes related to the breathing management protocol.

| Introduced failure mode | | PTV | | | | |
| --- | --- | --- | --- | --- | --- | --- |
| Planned | Delivered | Dmean (%) | Dmax (%) | D95% (%) | D98% (%) | D2% (%) |
| Expiration | Free-breathing | -38.5 | -2.7 | -96.1 | -95.5 | -3.4 |
|  | Inspiration | -72.5 | -6.0 | -97.1 | -96.0 | -8.5 |
|  | Gated 30-60 | -13.1 | -1.0 | -64.5 | -76.6 | -0.4 |
| Inspiration | Free-breathing | -43.4 | -1.6 | -97.4 | -96.3 | -2.0 |
|  | Expiration | -76.8 | -1.5 | -98.0 | -96.6 | -3.7 |
|  | Gated 30-60 | -62.7 | -0.4 | -97.9 | -96.5 | -2.3 |
