# Supplementary figures and images for: Determining optimal transit dosimetry gamma parameter values for the detection of failure modes using receiver operating curve analysis
Source: J Appl Clin Med Phys. 2025 Dec 29;27(1):e70424. doi: 10.1002/acm2.70424 (PMC12746048; doi:10.1002/acm2.70424)

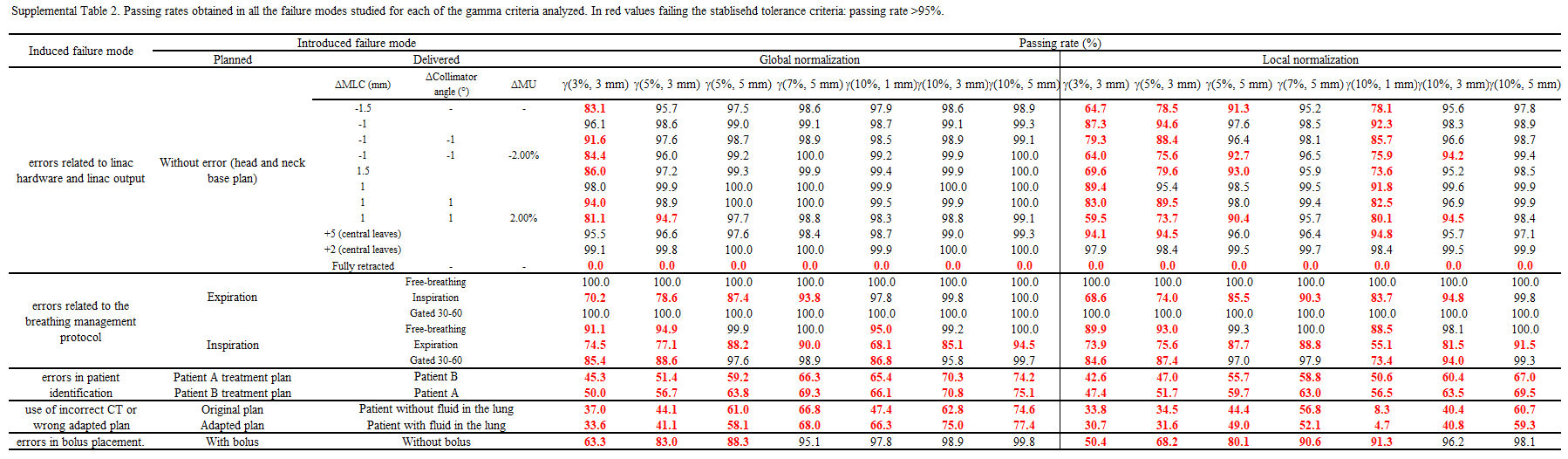

Supplement: Supplementary file 3 — Supporting Information [file ACM2-27-e70424-s001.png]
